# Notes and Comments

**Published:** 1897-03

**Authors:** 


### Notes and Comments.[1]

Are We all at Sea?—In following closely the investigations of Dr. G. V. Black upon the "Physical Characteristics of the Teeth," contributed to the *Dental Cosmos*, one would naturally ask the question, "Are We all at Sea?" Are we to believe, accept, and act upon Dr. Black's most important conclusions? Some of these are that caries of the teeth is not dependent upon the condition of the tissues of these organs, but upon the condition of their environment; that there is no basis for the supposition that some teeth

[1] The assistant editor solicits contributions for this department,—new methods, new remedies and formulas, or any short practical note which may prove of value to the practitioner or student. Address 1718 Walnut Street, Philadelphia.

are too soft or too poorly calcified to bear filling with gold; no basis for the supposition that the teeth of children under the age of twelve are too soft to receive such fillings; no basis for the selection and adaptation of filling materials to soft, hard, or poorly calcified teeth; the only basis for the selection of filling materials is the operator's judgment as to which *he* can most perfectly manipulate. Also that there is no basis for the supposition that pericemental inflammation, or pyorrhœa, attacks dense teeth any more than those less dense. Come forward, histologists, microscopists, and pathologists, and tell us,—Are we all practising and teaching such radical errors? Are we all at sea?

---

"Platir."—Dr. W. Storer How, in writing upon alloys in the *Dental Cosmos*, says, "An alloy of platinum and iridium has long been in use by dentists in plate, wire, and other forms, and a brief designation of the alloy has hence become a desideratum." Iridio-platinum, or platinum and iridium, he says, have hitherto been the cumbersome terms used for indicating the compound metal, and in the interest of economy, brevity, and perspicacity the term *platir* is suggested. Platir plate, platir wire, platir posts, etc., would therefore be used whenever those articles were desired to be composed of an alloy of platinum and iridium. Dr. How does not state what the term platir indicates, from what it is derived, or give any reason for adopting it.

There are certain words and phrases which convey certain definite ideas to our minds,—iridio-platinum and platinized gold are examples,—and when we are counselled to discard them we must have good and sufficient reasons for so doing.

---

Methods of Tooth-Bleaching.—A correspondent asks us to give "Dr. Kirk's method of bleaching teeth" through "Notes and Comments." Dr. Kirk has done much in the study of tooth-bleaching methods, giving us from time to time valuable information upon the subject, and has published two original methods for accomplishing this object,—viz., one in which sulphurous acid is evolved from a mixture of boracic acid and sodium sulphite, which is detailed in the "American System of Dentistry," and another one suggesting the use of sodium dioxide, reported in the *Dental Cosmos* of March, 1893. His more recent practice, however, is about as given in the following quotation.

We assume that our correspondent understands that the best results are accomplished when the products of decomposition in the dentinal tubuli are removed. This may be accomplished, as Dr. Kirk says in a paper before the Pennsylvania State Society, "either by combining with hydrogen dioxide a caustic alkali which will saponify and render soluble all fatty matter and organic *débris*, or by the use of sodium dioxide, a compound which combines in itself the properties of both hydrogen dioxide and a caustic alkali. For the former process I know of no better method than that devised by Dr. D. N. McQuillen, in which he precedes the application of pyrozone twenty-five-per-cent. by the use of Schreier's kalium-natrium preparation. This renders thoroughly soluble the contents of the pulp-chamber, canal, and tubuli, which are afterwards bleached and mechanically cleansed by the chemical and effervescent action of the pyrozone. After a tooth has been thus bleached its structure should be saturated with some indestructible material to prevent subsequent ingress of matters which may cause rediscoloration. For this purpose I use a lacquer which consists of a solution of pyroxylin in methyl alcohol, known as "kristaline."

---

"A Blot on the Profession."—Dr. W. H. Trueman, in writing upon the subject, says there is in the leading paper of the December (1895) number of the *Dental Digest* a point which, to his mind, especially invites criticism. It is this,—and it is a fault far too common in our profession,—the gross, if not inexcusable, carelessness in the use of assumed facts upon which to base disparaging remarks regarding a professional brother. The writer makes a grave accusation upon either the intelligence or the integrity of men of good repute and of recognized skill, based upon the bare statements of patients who were formerly under their care. What evidence had he that those statements were true? What evidence that they were not, unintentionally perhaps, misleading? So far as the article shows, he made no efforts and exercised no care to verify the statements made, but at once pronounced judgment that the three operators—whose acknowledged professional attainments, if a well earned professional reputation is worth anything, fully entitled them to a hearing—were guilty of either ignorance or indifference regarding a most important question.

